# Pubertal Timing and Growth Dynamics in Children With Severe Primary IGF-1 Deficiency: Results From the European Increlex^®^ Growth Forum Database Registry

**DOI:** 10.3389/fendo.2022.812568

**Published:** 2022-02-18

**Authors:** Peter Bang, Michel Polak, Valérie Perrot, Caroline Sert, Haris Shaikh, Joachim Woelfle

**Affiliations:** ^1^ Division of Paediatrics, Department of Biomedical and Clinical Sciences, Linköping University, Linköping, Sweden; ^2^ Paediatric Endocrinology, Gynaecology and Diabetology, Centre de Référence des Maladies Endocriniennes Rares de la Croissance, Hôpital Universitaire Necker Enfants Malades, AP-HP, Université de Paris, Paris, France; ^3^ Ipsen Pharma, Boulogne-Billancourt, France; ^4^ Ipsen Pharma, Milton Park, United Kingdom; ^5^ Endocrinology and Diabetology, University Children’s Hospital, Friedrich-Alexander University Erlangen-Nürnberg, Erlangen, Germany

**Keywords:** growth retardation, severe primary insulin-like growth factor-1 deficiency, puberty, mecasermin, Eu-IGFD Registry

## Abstract

**Background:**

Puberty is delayed in untreated children and adolescents with severe primary IGF-1 deficiency (SPIGFD); to date, it has not been reported whether recombinant human insulin-like growth factor-1 mecasermin (rhIGF-1) treatment affects this. Pubertal growth outcomes were extracted from the European Increlex^®^ Growth Forum Database (Eu-IGFD) Registry (NCT00903110).

**Methods:**

The Eu-IGFD Registry includes children and adolescents aged 2 to 18 years with growth failure associated with SPIGFD who are treated with rhIGF-1. Reported outcomes include: age at last registration of Tanner stage 1 and first registration of Tanner stage 2-5 (T2-T5; based on breast development for girls and genital development for boys, respectively); maximum height velocity during each Tanner stage; and pubertal peak height velocity (PPHV). Data cut-off was 13 May 2019.

**Results:**

This analysis included 213 patients (132 boys and 81 girls). Mean (SD) age at last registration of T1 and first registration of T5 was 13.0 (2.0) and 16.3 (1.6) years, respectively, in boys and 11.6 (1.8) and 14.7 (1.5) years, respectively, in girls. Among patients reaching the end of puberty (25 boys and 11 girls), mean (SD) height SDS increased from -3.7 (1.4) at baseline in the Eu-IGFD Registry to -2.6 (1.4) at T5 in boys and from -3.1 (1.1) to -2.3 (1.5) in girls. Maximum height velocity was observed during T2 in girls and T3 in boys. Median (range) PPHV was 8.0 (0.3–13.0) cm/year in boys and 6.8 (1.3–9.6) cm/year in girls and occurred most frequently during T2. Overall, the adverse events seen in this analysis were in line with the known safety profile of rhIGF-1.

**Conclusion:**

Children and adolescents treated with rhIGF-1 for SPIGFD with growth failure experienced an increase in height SDS in prepubertal years compared with baseline. Despite 1.5 years delay in pubertal start and a delayed and slightly lower PPHV, height SDS gain during puberty was maintained.

## Introduction

The growth hormone (GH)/insulin-like growth factor-1 (IGF-1) axis is crucial for linear growth and pubertal growth promotion (1, 2), and IGF-1 deficiency causes severe growth retardation. Severe primary IGF-1 deficiency (SPIGFD) comprises a group of rare growth disorders, with a prevalence of approximately 1% within the spectrum of IGF-1 deficiency disorders (3). The GH/IGF-1 axis is disrupted in children with SPIGFD with GH insensitivity (low IGF-1 levels, despite normal or elevated GH secretion) (4, 5), leading to growth failure. Physical characteristics resulting from SPIGFD include extremely short stature, retarded organ growth, small hands and feet, and under-development and weakness of the muscular system (6, 7). Disruption to the GH/IGF-1 axis is also thought to have a negative impact on gonadal function and pubertal development in patients with SPIGFD (8–11).

Growth failure associated with SPIGFD in children aged 2 to 18 years can be successfully treated, especially when diagnosed early (12), with long-term administration of recombinant human IGF-1 (rhIGF-1). The rhIGF-1 Increlex^®^ (mecasermin [rDNA origin]; Ipsen Pharma, France) has been licensed for the treatment of SPIGFD since 2005 in the USA and 2007 in Europe (4, 5). Clinical trials have demonstrated that rhIGF-1 stimulates linear growth in children with SPIGFD, leading to increased height velocity (13, 14). Despite being used in clinical practice for over a decade, the impact of rhIGF-1 treatment on pubertal growth dynamics has not been extensively assessed. The effect of rhIGF-1 on pubertal development was described as part of a study assessing the safety and efficacy of rhIGF-1 in children with short stature and low IGF-1 levels, which showed that pubertal development occurred at appropriate ages in all individuals, except one; however, patient numbers in this study were low (15). Two important characteristics of the growth spurt at puberty are the pubertal peak height velocity (PPHV) and the age at which the PPHV occurs (16, 17); as the effect of rhIGF-1 treatment on these variables is currently unknown, and further research is required.

The European Increlex^®^ Growth Forum Database (Eu-IGFD) Registry is an ongoing, open-label observational study to monitor the long-term safety and effectiveness of rhIGF-1 treatment in children and adolescents with growth failure in routine clinical practice. The Registry aims to monitor patients during, and after the end of treatment and to the attainment of near adult height (1). Here, we describe pubertal growth dynamics in children and adolescents with SPIGFD with growth failure who were treated with mecasermin and whose data were entered into the Eu-IGFD.

## Materials and Methods

### Trial Design

The Eu-IGFD Registry is a descriptive, multicentre, observational, prospective, open-ended, non-interventional, post-authorization surveillance study (ClinicalTrials.gov ID: NCT00903110) conducted in ten European countries (Austria, Belgium, France, Germany, Italy, the Netherlands, Poland, Spain, Sweden and the UK), and initiated in December 2008.

The primary objective of the Eu-IGFD Registry is to collect long-term safety data on the use of mecasermin (rhIGF-1) for the treatment of children and adolescents with growth failure, including SPIGFD. The main secondary objective is to collect long-term effectiveness data for rhIGF-1 treatment in children and adolescents with growth failure. The Registry design has been described previously (1). The analysis presented in this manuscript covers data collected up to 13 of May 2019 and focuses on the effect of rhIGF-1 treatment on pubertal growth dynamics.

### Patients

The Eu-IGFD Registry includes children and adolescents aged 2 to 18 years. All children and adolescents presenting at participating centres with growth failure associated with SPIGFD, for whom rhIGF-1 is indicated, and those who are already receiving treatment with rhIGF-1, are eligible for enrolment into the Registry and are assessed throughout their course of treatment (irrespective of subsequent treatment changes). The decision to prescribe rhIGF-1 treatment is made independently of the decision to enrol the patient into the Registry (1). Children and adolescents currently participating in either a mecasermin clinical trial or in any clinical trial for treatment of growth retardation were excluded from the Eu-IGFD Registry.

The analysis presented in this manuscript includes children and adolescents who were prepubertal (Tanner stage [T] 1; before breast development in girls and genital development in boys) at first rhIGF-1 intake in the Eu-IGFD Registry, were not receiving gonadotropin-releasing hormone (GnRH) agonist treatment and whose data were entered in the Eu-IGFD Registry before 13 May 2019.

### Treatment

The administered dose of rhIGF-1 was in accordance with the European Summary of Product Characteristics (SmPC) for mecasermin (4) and local clinical practice. Dosing was individualised based on the treating-physician’s clinical judgment. According to the mecasermin prescribing information, doses of 0.04–0.12 mg/kg bodyweight are given twice daily by subcutaneous injection before or shortly after a meal or snack. The timing and dose of rhIGF-1 treatment were at the discretion of the treating-physician and were independent of the decision to include patient data in the Eu-IGFD Registry.

### Outcomes

Anonymous data in the patients’ medical records are collected using an electronic case report form. General methodology for the Eu-IGFD Registry and information collected at baseline (or the visit closest to the start of rhIGF-1 treatment) and each follow-up visit has been described previously (1). The number and frequency of follow-up visits are determined by the investigator’s judgment based on clinical need and mecasermin SmPC recommendations (1).

The following endpoints are reported in this manuscript: breast development in girls and genital development in boys were assessed at each visit according to Tanner stage, and the last registration of T1 and first registration of stages T2 to T5 were identified. Data were collected until patients reached adult height. In reporting the data on Tanner stage, an informal comparison was made with reference data from a healthy population in Denmark (18). Pubertal duration was defined as the time between last registration of T1 to first registrations of T4/T5. Maximum height velocity during each Tanner stage was calculated. PPHV was defined as the maximum annualised height velocity between two visits ≥6 months apart during T2 to T4/T5. The Tanner stage at which PPHV occurred was noted. The evolution in height SDS during pubertal development was also assessed.

Adverse events (AEs) were reported by the investigator and classified as serious or non-serious, as mild, moderate or severe, and whether they were related or not to rhIGF-1 treatment. Neoplasia events and all ‘targeted’ AEs were collected. Targeted AEs are defined as those AEs that were shown to occur frequently or historically associated with rhIGF-1 treatment (i.e., headache, otitis media, papilledema, hypoglycaemia, acromegalic facial changes, oedema, gynaecomastia, hearing loss, intracranial hypertension, lipohypertrophy at injection site, myalgia, sleep apnoea, tonsillar hypertrophy, and cardiomegaly).

### Statistics

Descriptive statistics were used for all endpoints. Results are presented as mean (standard deviation [SD] or two-sided 95% confidence interval [CI] of the mean) and median (range or 25^th^ and 75^th^ percentiles). For categorical variables, the 95% CIs of the proportion are provided. Unless specified, continuous variables are given as median (range).

## Results

### Patient Characteristics

Between December 2008 and May 2019, 281 patients were enrolled in the Eu-IGFD Registry; of these: 213 (132 boys and 81 girls) were prepubertal and were included in this analysis (**Table 1**); 157 (73.7%) were treatment-naïve (i.e., had not received previous growth-promoting treatment); and SPIGFD was the diagnosis in 188 (88.3%) patients (**Table 1**). All patients who were pubertal at the start of rhIGF-1 treatment in the Registry have been excluded from this analysis.

**Table 1:** Patient characteristics of prepubertal^a^ patients at the start of rhIGF-1 intake in the Eu-IGFD Registry (baseline) and at last rhIGF-1 intake.

	Boy (n=132)	Girl (n=81)	Total (N=213)
**Patient characteristics at the start of rhIGF-1 intake (baseline)**			
Previously treated, n (%)^b^	37 (28.0)	19 (23.5)	56 (26.3)
Treatment-naïve, n (%)	95 (72.0)	62 (76.5)	157 (73.7)
Age, years			
Mean (SD)	8.8 (3.8)	8.1 (3.6)	8.6 (3.7)
Median (range)	8.6 (0.4–16.1)	8.2 (1.9–14.8)	8.3 (0.4–16.1)
Height, cm			
n	117	74	191
Mean (SD)	111.3 (20.0)	106.8 (20.8)	109.6 (20.4)
Height SDS			
n	117	74	191
Mean (SD)	-3.7 (1.4)	-4.0 (1.4)	-3.8 (1.4)
BMI SDS			
n	105	68	173
Mean (SD)	-0.7 (1.4)	-0.9 (1.3)	-0.8 (1.4)
Bone age, years			
n	23	16	39
Mean (SD)	7.8 (3.2)	7.1 (3.1)	7.5 (3.2)
Height velocity, cm/y			
n	75	41	116
Mean (SD)	4.6 (1.7)	5.1 (1.8)	4.8 (1.8)
Diagnosis, n (%)^c^			
Severe primary IGF-1 deficiency	116 (87.9)	72 (88.9)	188 (88.3)
Primary IGF-1 deficiency	9 (6.8)	7 (8.6)	16 (7.5)
GH gene deletion with anti-GH antibodies	1 (0.8)	0 (0.0)	1 (0.5)
Small for gestational age	2 (1.5)	2 (2.5)	4 (1.9)
Insulin resistance syndrome	0 (0.0)	1 (1.2)	1 (0.5)
Diabetes	1 (0.8)	0 (0.0)	1 (0.5)
Other	6 (4.5)	2 (2.5)	8 (3.8)
Laron syndrome, n (%)	18 (13.6)	12 (14.8)	30 (14.1)
**Patient characteristics at last rhIGF-1 intake** **^d^**			
Age, years			
Mean (SD)	12.9 (4.0)	11.6 (3.6)	12.4 (3.9)
Median (range)	13.0 (2–22)	12.0 (4–18)	12.6 (2–22)
Pubertal stage at last visit while on treatment, n (%)	115	75	190
1	56 (48.7)	38 (50.7)	94 (49.5)
2	14 (12.2)	10 (13.3)	24 (12.6)
3	11 (9.6)	9 (12.0)	20 (10.5)
4	19 (16.5)	12 (16.0)	31 (16.3)
5	15 (13.0)	6 (8.0)	21 (11.1)
Missing data	17	6	23
Height, cm			
n	131	79	210
Mean (SD)	135.1 (22.4)	128.0 (19.7)	132.4 (21.7)
Height SDS			
n	131	79	210
Mean (SD)	-2.9 (1.5)	-3.0 (1.5)	-2.9 (1.5)
BMI SDS			
n	131	79	210
Mean (SD)	-0.1 (1.5)	-0.4 (1.4)	-0.2 (1.5)
Bone age, years			
n	34	16	50
mean (SD)	10.2 (4.4)	10.6 (3.3)	10.3 (4.0)
Height velocity, cm/y			
n	88	51	139
Mean (SD)	5.3 (2.3)	4.7 (2.0)	5.1 (2.2)

a Prepubertal patients not treated with a gonadotropin-releasing hormone agonist.

b In prepubertal patients followed until the end of puberty (excluding patients treated with gonadotropin-releasing hormone agonist), 14 were non naïve, including 11 previously treated with rhGH, 2 previously treated with rhIGF-1, and 1 previously treated with both rhGH and rhIGF-1.

c More than one diagnosis is possible.

d Or the time of evaluation if treatment with rhIGF-1 was ongoing.

GH, growth hormone; IGF-1, insulin-like growth factor-1; SD, standard deviation; SDS, standard deviation score.

Of the 36 participants assessed until the end of puberty (excluding patients treated with gonadotropin-releasing hormone agonist), 14 were non-naïve, including 11 who were previously treated with rhGH, 2 who were previously treated with rhIGF-1, and 1 who was previously treated with both rhGH and rhIGF-1.

The median (range) duration of follow-up from the start of rhIGF-1 treatment was 4.3 (0.2–11.0) years. Mean rhIGF-1 doses remained stable as puberty progressed, with median doses of 120 µg/kg bid at all stages of puberty. At 1 year after initiation of rhIGF-1 treatment (after the titration period), 107 (50.2%) were receiving 120 µg/kg bid (the recommended maximum dose) (4) or above (only 6 patients were receiving a dose above 120µg/kg bid), 24 (11.3%) were receiving 100-120 µg/kg bid, and 82 (38.5%) were receiving <100 µg/kg bid.

### Puberty and Pubertal Growth Dynamics

**Figure 1:**
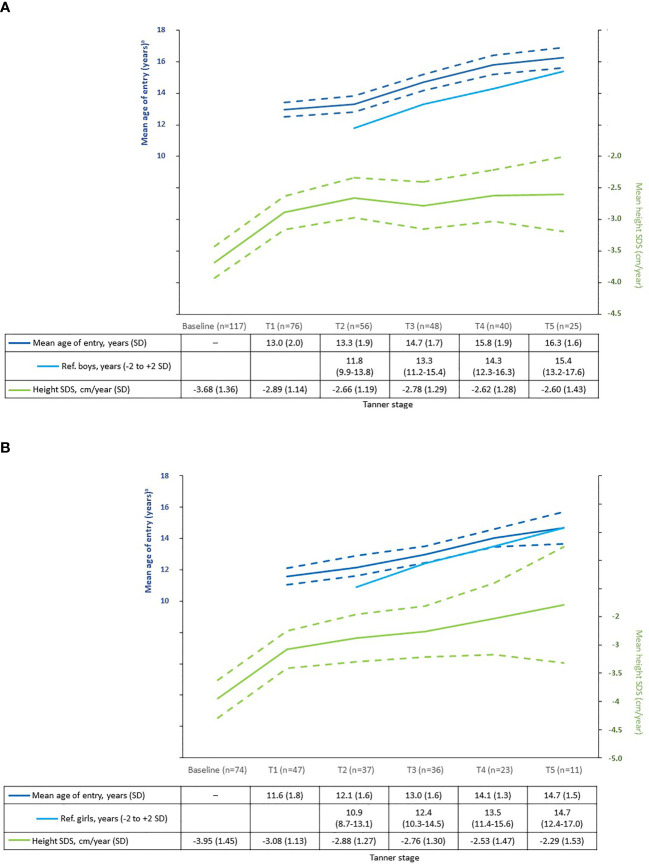
Mean age of entry into Tanner stage* and mean height SDS at each Tanner stage in rhIGF-1-treated children compared with reference population (18). **(A)** Boys. **(B)** Girls. Reference population: healthy Caucasian children from public schools in Denmark between 1991–1993. A total of 826 boys and 1100 girls (aged 6.0 to 19.9 years) were included. Dashed lines show 95% confidence intervals for the Eu-IGFD Registry population. *Except T1 values, which are age at last T1. ^a^For children in the Eu-IGFD Registry, this was the mean age at first registration into each Tanner stage. SPIGFD, severe primary insulin-like growth factor-1 deficiency; SD, standard deviation; SDS, standard deviation score.

The mean (SD) age at start of rhIGF-1 treatment for patients reaching the end of puberty was 10.9 (2.56) years for boys, and 9.1 (1.83) years for girls. The mean age of patients at entry into each Tanner stage is shown in **Figure 1** (not all children had visits at every Tanner stage as the time between clinic visits varied). Compared with a Danish reference population of healthy children (18), boys and girls with SPIGFD had delayed entry into T2, with approximately 1.5 years, and with less delayed entry into T4/T5. Among the patients reaching the end of puberty (T5; 25 boys and 11 girls), mean (SD) pubertal duration from last T1 was 3.7 (1.2) years in boys and 3.9 (1.0) years in girls. During pubertal development, height SDS was unchanged in boys while an apparent increase was observed in girls (**Figure 1**).

Height SDS at T1-T5 for boys and girls is shown in **Table 2**. There was no correlation, in the small subgroup reaching the end of puberty (25 boys and 11 girls), between the duration of treatment in the prepubertal period and total height SDS gain or height SDS gain during the pubertal period.

**Table 2:** Height SDS at different Tanner stages of the subgroup of children receiving rhIGF-1 who reached the end of puberty during the time period of this analysis^a^.

	Boys (n=25)						Girls (n=11)					
	BL	T1	T2	T3	T4	T5	BL	T1	T2	T3	T4	T5
Age in years at Tanner stage, mean (range)	10.9 (5.8 to 15.3)	12.6 (8.0 to 16.0)	13.1 (8.6 to 16.3)	14.2 (11.0 to 16.5)	15.2 (11.9 to 17.4)	16.3 (12.3 to 19.0)	9.1 (6.1 to 11.2)	10.8 (8.8 to 13.0)	11.5 (9.5 to 13.7)	12.4 (10.6 to 15.9)	13.4 (11.0 to 15.4)	14.7 (12.0 to 17.4)
Height SDS, mean (range)	-3.7 (-7.0 to -1.7)	-3.1 (-3.7 to -2.4)	-2.9 (-6.1 to -1.3)	-2.8 (-6.6 to -0.8)	-2.9 (-7.0 to -1.2)	-2.6 (-6.9 to -0.5)	-3.1 (-5.9 to -2.0)	-2.7 (-3.1 to -2.1)	-2.6 (-4.6 to -1.1)	-2.3 (-5.1 to -1.1)	-2.3 (-6.1 to -0.9)	-2.3 (-6.5 to -1.0)

a at latest registration of T1, and at first registration of T2, T3, T4 and T5.

BL, baseline; SDS, standard deviation score.

**Figure 2:**
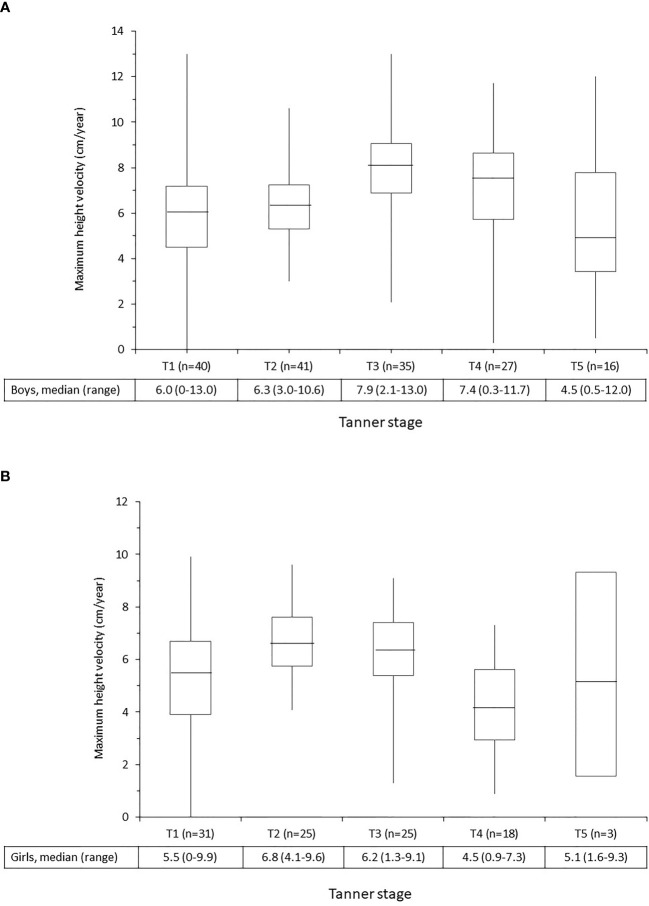
Maximum height velocity at each Tanner stage in children receiving rhIGF-1 for growth failure. **(A)** Boys. **(B)** Girls. The middle box represents the interquartile range; the mid-line represents the median value. The upper/lower whiskers represent the upper and lower quartiles. SPIGFD, severe primary insulin-like growth factor-1 deficiency.

**Figure 3:**
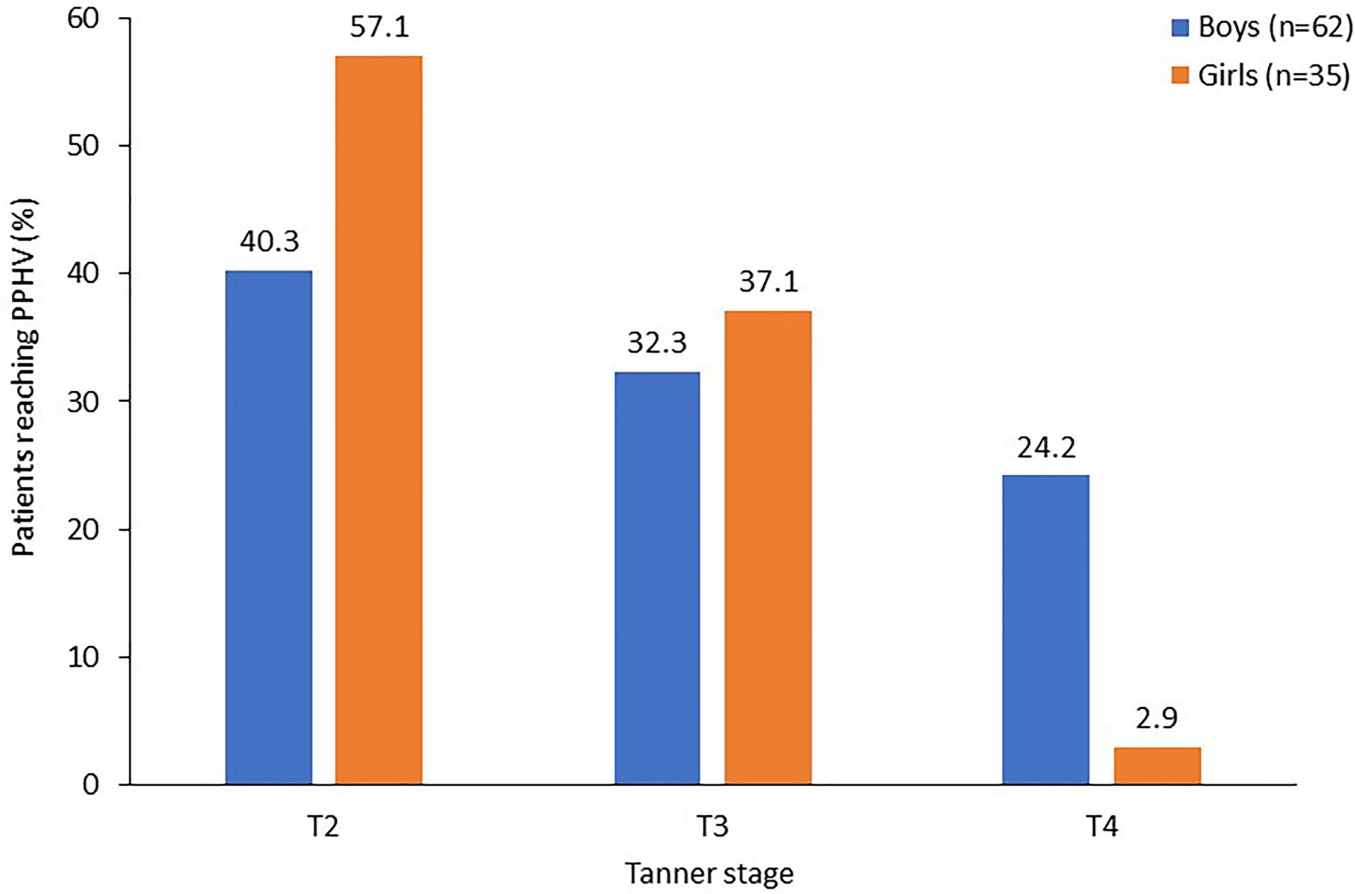
Pubertal stage at pubertal peak height velocity in children receiving rhIGF-1 for SPIGFD with growth failure. Percentage of patients who started puberty with available data on PPHV, and stage at which PPHV occurred. Percentages for each gender do not add up to 100% as Tanner stage 5 has been omitted from this figure. PPHV, pubertal peak height velocity; SPIGFD, severe primary insulin-like growth factor-1 deficiency.

Maximum height velocity was achieved in T2 (breast development) in girls and in T3 (genital development) in boys (**Figure 2**). For the overall period of puberty, median (range) PPHV was 8.0 (0.3–13.0) cm/year in boys (n=62) and 6.8 (1.3–9.6) cm/year in girls (n=35). PPHV was observed at T2 for 40% of boys and 57% of girls (**Figure 3**) and mean (SD) age at PPHV was 15.3 (1.9) years in boys (n=62) and 13.3 (1.8) years in girls (n=35). In the subgroup of patients reaching T4/T5 at the time of this analysis, mean (SD) PPHV was 8.2 (2.3) cm/year in boys (n=43) and 7.2 (1.5) cm/year in girls (n=23).

Mean (SD) growth recorded between the last registration of T1 and first registration of T4 was 17.0 (6.4) cm in boys (n=40) and 17.5 (3.8) cm in girls (n=23), and between the last registration of T1 and first registration of T5 was 25.2 (7.2) cm in boys (n=24) and 20.8 (3.9) cm girls (n=11).

### Safety

In this population of 213 patients who were prepubertal at the time of initiation of rhIGF-1 treatment, 143 (67.1) had at least one treatment-emergent AE (TEAE; **Table 3**). The three most frequent TEAEs were: hypoglycaemia, 23.9%; lipohypertrophy, 11.7%; and headache, 11.7% (**Table 3**). Targeted TEAEs were reported in 109 patients (51.2%), and 15 patients (7.0%) had 25 serious targeted TEAEs. Twenty-two patients (10.3%) had 39 serious TEAEs that were considered, by the investigator, to be related to treatment. Neoplastic TEAEs were reported in six patients: two cases of melanocytic naevus, and one each of dysplastic naevus, haemangioma of skin, myelodysplastic syndrome and papillary thyroid cancer. Eleven patients (5.2%) withdrew because of TEAEs. Two patients (0.9%) had a fatal TEAE: one patient had myelodysplastic syndrome; and one patient had a complication of a bone marrow transplant. In both patients there were other confounding medical conditions and death was considered unrelated to the study drug by the reporting investigators.

**Table 3:** Overview of frequently reported (≥2% of patients) treatment-emergent adverse events in children receiving rhIGF-1 with growth failure^a^.

	Number of patients (%)^b^ (N = 213)
Any TEAE	143 (67.1)
Serious TEAE	47 (22.1)
Treatment-related TEAE	107 (50.2)
Targeted TEAE	109 (51.2)
**Most frequent TEAEs (≥2% of patients)**	
Hypoglycaemia	51 (23.9)
Lipohypertrophy	25 (11.7)
Headache	25 (11.7)
Tonsillar hypertrophy	22 (10.3)
Otitis media	17 (8.0)
Insulin-like growth factor increased	12 (5.6)
Deafness	8 (3.8)
Adenoidal hypertrophy	6 (2.8)
Injection site pain	6 (2.8)
Acromegaly^c^	6 (2.8)
Sleep apnoea	5 (2.3)

a Median duration of follow-up from the start of rhIGF-1 treatment, 4.3 years.

b With at least one event.

c Acromegalic facial changes

TEAE, treatment-emergent adverse event; TSH, thyroid stimulating hormone.

## Discussion

The results from this analysis of Eu-IGFD Registry population of rhIGF-1-treated patients with SPIGFD and growth failure suggest that puberty is delayed by approximately 1.5 years and that PPHV is delayed in both sexes despite ongoing rhIGF-1 treatment. Treatment with rhIGF-1 may provide improvements in measures of pubertal growth dynamics, including maintenance of (boys) or slight further increase (girls) in height SDS during puberty. The total pubertal height gain in the limited number of patients with SPIGFD reaching T5 during the time period of this analysis was within the expected range for healthy boys and girls, respectively, as was the duration of puberty (last T1 through to T5) (18). The dose of rhIGF-1 may be of importance, and for safety reasons should not exceed 120 µG/kg bid (4), nevertheless in this analysis, not all of the patients received the recommended maximum dose of rhIGF-1. However, the responsiveness to rhIGF-1 is likely to be individual and we have previously failed to identify an rhIGF-1 dose that can predict the first year height response in patients with or without Laron syndrome (12).

Data on age of entry into Tanner stages were compared with a reference population (consisting of 826 and 1100 healthy Caucasian boys and girls, respectively), aged 6.0 to 19.0 years, from public schools in Denmark between 1991 and 1993 (18). These normative data were used because they provide reliable information from a large European population sample. When compared with this reference population, the Eu-IGFD Registry population started puberty approximately 1.5 years later. Laron et al. (19) described reference values for untreated children with SPIGFD, in which the authors noted that puberty was more delayed in boys than in girls: the mean onset of puberty in girls with Laron Syndrome was 10.7 (0.7) years and 15.6 (2.6) years in boys with Laron Syndrome (compared with 12.1 years and 13.3 years, respectively, in the rhIGF-1-treated Eu-IGFD population). Thus rhIGF-1 treatment of patients with SPIGFD does not appear to completely correct the age at which puberty occurs.

The population in the Eu-IGFD Registry reported here reached a maximum height velocity later in life than historical healthy controls [15.2 years vs approximately 13.5 years for boys, respectively (20); 13.3 years vs approximately 11.5 years for girls, respectively (20)]. However, untreated children with SPIGFD lack the typical pubertal growth spurt usually seen in children without GH insensitivity (19), and therefore, in the Eu-IGFD Registry population, rhIGF-1 treatment may restore, to a certain extent, the pubertal growth spurt compared with no treatment. Nevertheless, further research is needed to confirm these findings. PPHV was approximately 2 cm/year lower in patients with growth failure included in the Eu-IGFD Registry than in healthy populations (20).

The AE profile reported in this analysis of the Eu-IGFD Registry is generally consistent with previous reports of AEs during long-term treatment with rhIGF-1 (6). rhIGF-1 treatment may increase the risk of benign and malignant neoplasia in patients with SPIGFD (4, 21); therefore, special consideration of these events in this Registry population is important. Although available data do not allow calculations of relative risk, the current analyses included six neoplasm TEAEs (2.8% of the population). In those who receive rhIGF-1 treatment for unapproved uses or at above the recommended doses, risk of neoplasia may be higher. Clinicians should be vigilant for potential malignancy symptoms and if neoplasia develops, rhIGF-1 treatment should be discontinued, and appropriate expert medical care sought. However, the data in this study do not raise any new safety concerns.

While the Eu-IGFD Registry is a robust source of long-term data in a large Europe-wide population, an updated analysis of the data would provide a larger dataset for analysis of near adult height and pubertal growth characteristics. Other limitations in these data stem from the non-interventional nature of the Eu-IGFD Registry. For example, the frequency of visits to physicians may have resulted in some stages of puberty being unrecorded. There are also insufficient data on patients who stopped treatment before puberty. As is typical of registries, there is no comparator group and the use of previously published populations (e.g., from Denmark and the UK) may be sub-optimal, but studies of healthy children across the same geographical range as the Eu-IGFD are lacking. Furthermore, it was not possible to establish representative control populations as the ethnicity, country of origin and immigrant status of the study population were not routinely collected in the Registry. Nevertheless, while this represents a drawback of the current analysis, the use of a control group originating from Europe and inclusion of comparator populations large enough to be considered reliable may mitigate these methodological limitations to some extent. In previous analyses of height data from the Eu-IGFD Registry, we focused on children who were prepubertal and naïve to treatment that may affect growth. In the current analysis, most patients (157 of 213) were treatment naïve, but importantly 14 of the 36 children who reached the end of puberty had received prior growth-promoting therapy, including 11 previously treated with rhGH, 2 previously treated with rhIGF-1, and 1 previously treated with both rhGH and rhIGF-1. We do not yet have data regarding the first-year height response in patients previously treated with growth-promoting therapy compared with treatment-naïve patients, but responses may be lower than in treatment-naïve patients. This means there is a potential risk of underestimating the first-year height response in the group of children that reached T5, and were prepubertal at start of rhIGF-1. It is worth noting here that rhIGF-1 is approved for patients with SPIGFD and GH sufficiency, and therefore, is not considered a reasonable alternative to rhGH treatment in GH-sensitive patients. Despite these limitations, these data are the first of their kind, and therefore do add to our knowledge on the impact of rhIGF-1 on pubertal growth dynamics.

The results from this analysis provide further support to the concept that the GH/IGF-1 axis has a crucial role in gonadal function and pubertal development. While there has been a lack of direct evidence showing the benefit of IGF-1 treatment on pubertal development in patients with SPIGFD, indirect evidence has come from studies in patients with GH insensitivity syndrome, which offer a unique human model to study the effects of congenital IGF-1 deficiency. In these patients, pubertal development is delayed and genitalia and gonads are typically small (8, 9). Furthermore, findings from *in vivo* and clinical studies have demonstrated the importance of IGF-1 in supporting testicular function and steroidogenesis (10, 11).

In conclusion, boys and girls treated with rhIGF-1 for SPIGFD with growth failure experienced an increase in height SDS compared with baseline. rhIGF-1-treated patients entered puberty at an older age than children in a previously reported healthy population; and PPHV was achieved later in life and was lower overall than in a previously reported healthy population. Despite an older age at pubertal start, rhIGF-1 treated children with SPIGFD maintain or slightly increase their height SDS during pubertal years. Current knowledge of IGF-1 biology indicates that IGF-1 could play a role in malignancies in all organs and tissues. Physicians should therefore be vigilant of any symptoms of potential malignancy. If benign or malignant neoplasia develops, rhIGF-1 treatment should be discontinued, and appropriate expert medical care sought immediately. Overall, the AEs seen in this analysis were in line with the known safety profile of rhIGF-1. Data from this analysis suggest that, compared with no treatment, rhIGF-1 may provide improvements for children with growth failure due to SPIGFD.

## Eu-IGFD Registry Study Group

**Austria:** G. Hausler, K. Zwiauer; **Belgium**: M.C. Lebrethon, J. de Schepper; **France**: P. Adiceam, C. Braun, B. Cammas (previously M. Colle), H. Carla-Malpuech, C. Cessans, I. Cloix, M. Cogne, R. Coutant, M. de Kardenet, C. Gayet (previously E. Mallet), P. Hassler, M. Houang, A. Lienhardt, A. Linglart, F. M’Bou, M. Nicolino, F. Njuieyon, M. Petrus, M. Pinget, M. Polak, R. Reynaud, P.F. Souchon, M.T. Tauber, K. Wagner, J. Weill; **Germany:** I. Akkurt, S. Al Sawaf, S. Bechtold, M. Bettendorf, D. Bierkamp-Christophersen, J.-G. Blanke, H.-G. Doerr, A. Enniger (previously H. Leichter), M. Frühwald, K. Hartmann, B. Hauffa, E. Hammer, P.-M. Holterhus, A. Hübner, J. Ittner, C. Jourdan, A. Keller, H.S. Kim-Berger, B. Köster (previously Rosenbaum), J. Krüger (previously Richter-Unruh), C. Land, F. Lorenzen, T. Meissner, K. Mohnike, M. Morlot, H. Müller, C. Ockert, R. Oeverink, T. Rohrer, R. Pankau, C.-J. Partsch, E. Schönau, A. Schuster, K. Schwab, G. Simic-Shleicher, B. Tittel, K. Warnke (previously W. Bonfig), J. Wölfle; **Italy:** S. Cannavò, M. Cappa, V. Cherubini, G. Citro, D. Concolino, M-F. Fainza (previously L. Cavallo), P. Francesco Perri, L. Guazzarotti (previously G. Vincenzo Zuccotti), A. Lampis, S. Longhi, M. Maghnie, R. Minelli (previously S. Bernasconi), L. Perrone, A. Pilotta, A. Sinisi, G. Weber (previously G. Chuimello), S. Zucchini; **Netherlands**: A. Hokken-Koelega, **Poland:** I. Ben-Skowronek, D. Birkholz, A. Bossowski, M. Hilczer, M. Korpal-Szczyrska, J. Smyczynska, L. Szewczyk; **Spain:** J. Argente, C. Bezanilla, M.-F. Borras, A. Carrascosa, R. de Sotto, R. Diaz, A. Feliu-Rovira, C. Fernandez, M. Ferrer, E. Gallego, F. Hermoso, A. Lechuga-Sancho, C. Luzuriaga, J. Martos, M-F. Moreno-Macian, P. Prieto, C. Rodriguez, J. Sanchez Del Pozo, A. Vela; **Sweden:** P. Bang, K. Ekström, N.-Ö. Nilsson; **United Kingdom:** F. Ahmed, L. Denvir, H. Johnstone, T. Mushtaq, L. Patel, C. Peters (previously K. Hussain), R. Ramakrishnan, S. Rose, N. Shaw, H. Storr.

## Data Availability Statement

The original contributions presented in the study are included in the article/supplementary material. Further inquiries can be directed to the corresponding author. Where patient data can be anonymised, Ipsen will share all individual participant data that underlie the results reported in this article with qualified researchers who provide a valid research question. Study documents, such as the study protocol and clinical study report, are not always available. Proposals should be submitted to DataSharing@Ipsen.com and will be assessed by a scientific review board. Data are available beginning 6 months and ending 5 years after publication; after this time, only raw data may be available.

## Ethics Statement

The ongoing Eu‑IGFD Registry is being conducted in compliance with independent Ethics Committees/Institutional Review boards (except in the UK, where ethical review is not required for this type of registry), informed consent regulations, the Declaration of Helsinki, the International Conference on Harmonization, and the Good Epidemiological Practice Guidelines. In addition, the Eu‑IGFD Registry adheres to all local regulatory requirements including data protection linked to the use of electronic data. Written informed consent was obtained from the parents or legal guardians and the patient (where applicable) before enrolment and data collection.

## Author Contributions

All authors contributed to the preparation of this manuscript. All authors reviewed and approved the final version of the manuscript. All authors have accepted responsibility for the entire content of this submitted manuscript and approved submission.

## Funding

This study was sponsored by Ipsen.

## Conflict of Interest

PB has attended advisory boards and received board of directors fees from Ipsen and Lilly, and has received consulting fees from Ipsen, Sandoz, Pfizer, Lilly and Versatis. JW has attended advisory boards and received board of directors fees from Ipsen and Novo Nordisk, and has received corporate-sponsored research fees from Pfizer and Ipsen, as well as speaker fees from Merck-Serono, Hexal, Pfizer and Novo Nordisk. MP has attended advisory boards and received board of directors fees from Ipsen (Increlex Registry), Novo Nordisk (Global Norditropin Advisory board), Pfizer France, and has received corporate-sponsored research fees from Ipsen, Novo Nordisk, Pfizer, Sandoz, Merck and Sanofi, as well as speaker fees from Novo Nordisk and Ipsen. VP and CS are both employees of Ipsen.

The authors declare that this study received funding from Ipsen. The funder contributed to the study design, data collection and analysis, decision to publish, and funded editorial support for preparation of the manuscript.

## Publisher’s Note

All claims expressed in this article are solely those of the authors and do not necessarily represent those of their affiliated organizations, or those of the publisher, the editors and the reviewers. Any product that may be evaluated in this article, or claim that may be made by its manufacturer, is not guaranteed or endorsed by the publisher.
